# A Localization Method for Underwater Wireless Sensor Networks Based on Mobility Prediction and Particle Swarm Optimization Algorithms

**DOI:** 10.3390/s16020212

**Published:** 2016-02-06

**Authors:** Ying Zhang, Jixing Liang, Shengming Jiang, Wei Chen

**Affiliations:** 1 College of Information Engineering, Shanghai Maritime University, Shanghai 201306, China; yingzhang@shmtu.edu.cn (Y.Z.); liangjixing501@163.com (J.L.); smjiang@shmtu.edu.cn (S.J.); 2 Department of Computer Science, Tennessee State University, Nashville, TN 37209, USA

**Keywords:** underwater wireless sensor networks, mobility patterns, localization, mobility prediction, particle swarm optimization

## Abstract

Due to their special environment, Underwater Wireless Sensor Networks (UWSNs) are usually deployed over a large sea area and the nodes are usually floating. This results in a lower beacon node distribution density, a longer time for localization, and more energy consumption. Currently most of the localization algorithms in this field do not pay enough consideration on the mobility of the nodes. In this paper, by analyzing the mobility patterns of water near the seashore, a localization method for UWSNs based on a Mobility Prediction and a Particle Swarm Optimization algorithm (MP-PSO) is proposed. In this method, the range-based PSO algorithm is used to locate the beacon nodes, and their velocities can be calculated. The velocity of an unknown node is calculated by using the spatial correlation of underwater object’s mobility, and then their locations can be predicted. The range-based PSO algorithm may cause considerable energy consumption and its computation complexity is a little bit high, nevertheless the number of beacon nodes is relatively smaller, so the calculation for the large number of unknown nodes is succinct, and this method can obviously decrease the energy consumption and time cost of localizing these mobile nodes. The simulation results indicate that this method has higher localization accuracy and better localization coverage rate compared with some other widely used localization methods in this field.

## 1. Introduction

The marine economy has witnessed rapid development recently, and marine rights and interests are receiving more and more attention in many countries, therefore research on UWSNs has developed quickly, providing basic technical support to many application fields, such as ocean environment monitoring, resource exploration, natural disaster warning and military defense [1]. Besides the basic characteristics of ordinary WSNs, like large-scale deployment and limited energy, underwater WSNs have some differences with terrestrial ones. First, underwater communication can only be realized by acoustic signals, which have a lower bandwidth and higher error rates. Second, the scale of node deployment in underwater environments is larger and the beacon nodes are sparser. In addition, because of water currents, there is the non-negligible node mobility which may cause frequent changes to the network topology [2,3].

The localization of mobile nodes is important and necessary for underwater sensor networks. In the marine military defense area, the identification and tracking of the intrusion objects must rely on the nodes’ location. It can also be used for animal tracking in the underwater environment, where the localization of mobile nodes can help researchers to determine marine animals’ locations and movements easily, and in particular mobile nodes can extend the tracking area and better realize real-time tracking. In marine environmental monitoring, the detection area is usually very large and dynamic. With random mobility, the sensor nodes could realize the monitoring of the whole area. In this process, the localization of mobile nodes is necessary for identifying where the polluted areas will be. In addition, the path-planning and the coverage optimization of the underwater nodes cannot be realized without the localization of the mobile nodes [4,5]. The information collection and transmission are realized by the sensor nodes, and the relevant data without location will make no sense, so the localization of mobile sensor nodes has gradually become a research hotspot and focus.

However, the characteristics of UWSN described above present great difficulties and challenges to the localization of underwater mobile nodes in a large-scale network. Acoustic communication has a bigger propagation delay, lower bandwidth and higher error rate compared to radio communication. This imposes more limits on any localization algorithm. The algorithms based on transmission of tremendous amounts of data or higher real-time communication requirements will not be applicable in this kind of circumstance. In addition, the mobility of nodes makes the topology change frequently. The algorithms designed for static networks need to run the localization procedure periodically to update the nodes’ location, which may cause more energy consumption for communication in an underwater environment. Moreover, the batteries of underwater sensor nodes can almost never be replaced and the energy is strictly limited, so under normal circumstances, it is difficult to achieve higher localization accuracy and localization coverage rate in an underwater environment [6,7].

**Figure 1:**
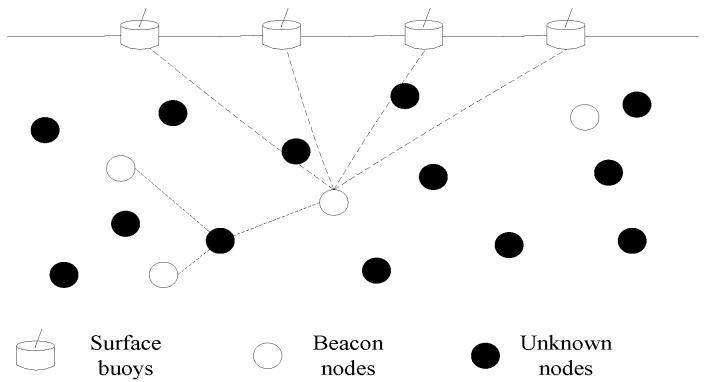
Schematic diagram of the monitoring model of an UWSN.

As shown in Figure 1, surface buoys equipped with GPS can obtain their locations. The number of beacon nodes is much less than the number of unknown nodes. Beacon nodes have more energy and their communication radius is about 200 m, so they can communicate directly with the buoys and have more neighbor nodes. In addition, beacon nodes have more hardware resources and better computing ability compared to unknown nodes, so they can use range-based localization methods. The unknown node are relatively cheaper and one does not want them to waste their energy. The communication radius of an unknown node is about 100 m and it cannot communicate with the buoys directly. Typically, it can only connect to its local (usually one-hop) neighbors. With local information exchange among themselves and nearby beacon nodes, the unknown nodes can achieve local positioning so as to effectively participate in the network activities.

Aiming at the special environment of UWSN and the difficulties in mobile node localization, we propose a method based on the mobility prediction and PSO algorithms. First we measure the distances between the beacon nodes and buoys, and locate the beacon nodes by using a range-based PSO algorithm which has a relative high accuracy. According to the location results at two time points, we can calculate the velocity of beacon nodes in the last instant. The velocities of the unknown nodes can be estimated by the velocity information of beacon nodes within the communication scope and the spatial correlation of underwater objects, and then the location of the unknown nodes for the next moment can be predicted by the mobility prediction method. Considering the higher energy consumption a the range-based algorithm, we just use it to locate the beacon nodes, whose number is far fewer compared to the unknown nodes, and on this basis, the localization of the unknown nodes can achieve a relatively high-accuracy result with fine instantaneity by the mobility prediction method. The rest of this article is organized as follows: in Section 2, some background knowledge and related work on nodes localization are introduced. Then, in Section 3, we describe the MP-PSO method in detail. Section 4 is the simulation and results analysis, and finally we conclude the paper in Section 5.

## 2. Related Works

Localization methods for wireless sensor networks can be divided into two types: range-based and range-free methods. The range-based methods such as the received signal strength indicator (RSSI), time difference of arrival (TDOA) and time of arrival (TOA) use hardware to measure the distance information. These kinds of method have a higher accuracy, but they increase the network cost and energy consumption [8]. The range-free methods use the connectivity of the network to locate the unknown nodes. The typical range-free methods mainly include the DV-HOP, Convex Programming and Centroid Localization algorithm. These methods have no additional hardware requirements, and they have lower energy consumption and shorter positioning time, but their accuracy is usually lower [9].

There are many researches on terrestrial nodes localization. In [10] the authors proposed a range-free localization algorithm based on a sequential Monte Carlo localization method. It can exploit mobility to improve the localization accuracy. In [11], a Monte Carlo localization algorithm with mobility prediction (MCL-MP) was proposed, and it can further improve the accuracy by using prediction and filtering for the unknown nodes based on dynamic sampling.

Studies on the localization of underwater mobile nodes always face some challenges, and most of them were designed for small-scale networks. For example, GPS Intelligent Buoys (GIB) based on surface buoys and one-hop communication under the water have been proposed. This approach has a high accuracy but the hardware is complex and the cost is high [12]. In [13], a so-called Silent Localization algorithm was proposed, which does not need time synchronization and is applicable to one-hop underwater networks. In addition, in [14], a Scalable Localization with Mobility Prediction (SLMP) method was proposed, and it is closer to localization in an actual environment. The SLMP algorithm has two stages to locate the unknown nodes. First, the velocity of beacon nodes will be estimated by the Durbin algorithm to perform the online linear prediction. The unknown nodes are then located by using the mobility prediction based on the spatial correlation of sensor nodes.

**Figure 2:**
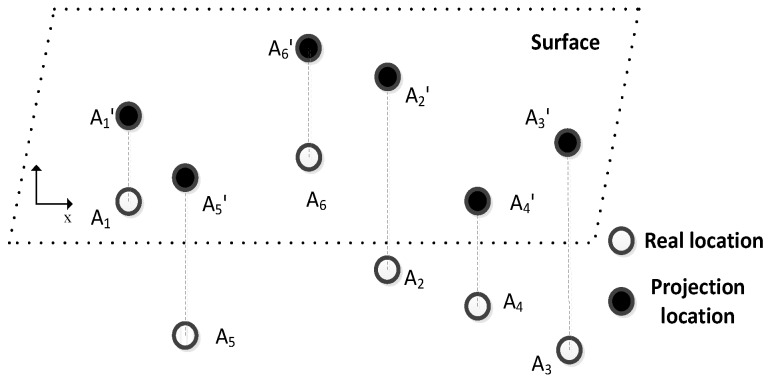
A schematic diagram of the projection method.

A three-dimensional deployment space is another important characteristic of UWSNs. In [15], an efficient localization scheme which can transform the three-dimensional localization problem into a two-dimensional counterpart via a projection technique was proposed; it can make all the nodes map to the same plane. Because the depth information can be obtained by a pressure sensor, this scheme not only makes the two-dimensional localization algorithm apply in the three-dimensional space, but also simplifies the amount of calculation for three-dimensional localization, and reduces the energy consumption of the process. In this paper, we also use this method to project the nodes on a certain plane, and only consider the velocity of the nodes in two orthotropic directions. The principle of this method is shown in Figure 2.

As shown in Figure 2, A_1_–A_6_ are sensor nodes in an underwater networks. We project them to the water surface and the corresponding locations are A_1_'–A_6_'. Then we only need to locate the nodes in the surface plane, and the final location can be obtained by simply adding the respective depth information.

## 3. MP-PSO Localization Method

The proposed MP-PSO method is a type of multi-step localization algorithm, and it can be divided into the beacon node localization and the unknown node localization. The beacon nodes are located by measuring the distances from the nodes to the buoys on the water surface with the range-based PSO algorithm, and it can achieve a higher localization accuracy. The locations of unknown nodes in the next moment can be predicted by estimating the speed of movement of the unknown nodes.

### 3.1. The Node Mobility Model 

Research shows that the mobility of underwater object is influenced by water current, temperature and some other factors [16], so we cannot use a unified model to describe the nodes mobility for all environments. However, under a specific environment, the nodes mobility is not completely random, so we can establish a specific mobility model for this specific environment according to the temporal and spatial correlation. Nowadays the UWSN is mainly used in seashore environment, where the water is relatively shallow, usually it is less than 100 m, and the water current is relatively flat, so the nodes’ mobility situation is not complex. In addition, the underwater nodes’ mobility in seashore area has an obvious characteristic, namely it demonstrates a certain semi periodic property because of tides [17].

**Figure 3:**
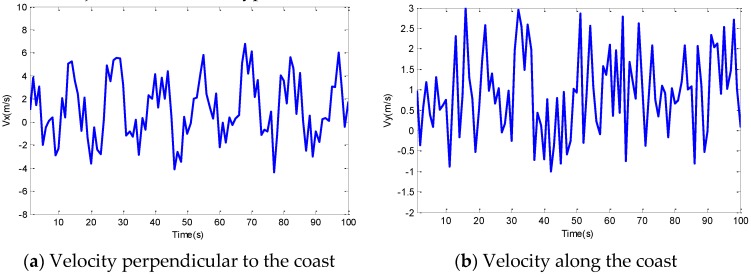
Mobility patterns of an underwater object in a seashore environment.

In [14], a mobility model based on the Euler algorithm was proposed, which takes the seashore environment as the application background. It assumes the flow velocity field is superposed by the tidal field and the remnant flow field. The tidal field is assumed to be oscillating homogeneously in one direction, and the remnant flow field is assumed to be an infinite sequence alternately rotating clockwise and anticlockwise by turns. The dimensionless velocity field in the kinematical model can be approximated as:

(1) $$Vx(t)=k_{1}\cdot \lambda \cdot v\cdot \sin(k_{2}\cdot x)\cdot \cos(k_{3}\cdot y)+k_{1}\cdot \lambda \cdot \cos(2k_{1}\cdot t)+k_{4}$$

(2) $$Vy(t)=-\lambda \cdot v\cdot \cos(k_{2}\cdot x)\cdot \sin(k_{3}\cdot y)+k_{5}$$

where *Vx* is the speed of the *X* axis direction and *Vy* is the speed of the *Y* axis direction, *λ* is the ratio of tidal excursion and the diameter of the remnant vortex. Here, *λ, k_1_, k_2_, k_3_* and *v* are related to factors like tides, temperature and salinity, and *k_5_* and *k_4_* are random variables. The values of these parameters are set in the simulation part of this paper. Figure 3 shows the velocity variation of an underwater object with time in a typical seashore environment.

This model is designed for the seashore environment, so it can evaluate the mobility of nodes whose velocity is relatively constant. As shown in Figure 3, the velocity in the two directions has an obvious semiperiodic property and temporal correlation, which can guarantee the practicability of mobility prediction of the unknown nodes’ localization in the water environment.

### 3.2. Propagation Loss Model for Submarine Sound Signal

In an underwater acoustic communication channel, the strength of a signal received by the unknown nodes: *S_r_* can be calculated as Equation (3). *S_t_* is the strength of the signal transmitted by beacon nodes. *A*(*d*,*f*) is the total attenuation for the acoustic signal in the transmission procedure:

(3) $$S_{r}=S_{t}-A(d,f)$$

Influenced by the environmental factors, the transmission attenuation *A*(*d*,*f*) is the sum of the extended losses and the absorption losses:

(4) $$A(d,f)=d^{\delta }\cdot \alpha^{d};\;\alpha =10^{\alpha (f)/10}$$

where, *d* is the transmission distance, *α* is the absorption coefficient, and δ is the attenuation coefficient (generally valued as 1 < δ < 2). *α*(*f*) is absorption loss, it can be approximately estimated based on Thorp algorithm [18], and it can be calculated as Equation (5):

(5) $$\alpha (f)=0.11\frac{f^{2}}{1+f^{2}}+44\frac{f^{2}}{4100+f^{2}}+2.75\times 10^{-4}f^{2}+0.003$$

where, *f* is the frequency of transmission signal of the nodes. Referring to the frequency of underwater network nodes widely used currently, the frequency can be set as 50 kHz, and the transmission power can be set as 2 W, namely 33 dBm [19].

### 3.3. Localization of Beacon Nodes

The swarm search strategy has the feature of adaptive optimization and the PSO algorithm is an efficient method which can realize accurate range-based localization for WSNs. The authors of [20] proposed a distributed node localization method on the basis of a multivariable optimization algorithm based on biogeography and the Particle Swarm Optimization algorithm, which can effectively improve the localization accuracy of the nodes. In [21], a binary Particle Swarm Optimization algorithm for distributed node localization in WSNs was proposed. Each unknown node performs localization by the measurement of distances from three or more neighbor beacon nodes to the unknown node. The nodes which already determined their locations will be regarded as the reference nodes for the residual nodes, and they will be involved in the subsequent localization procedure.

Considering the defect that the traditional PSO algorithm can easily fall into a local minimum and has lower convergence speed [22], in this paper, an inertia weight with Gaussian function decline and competition mechanism are adopted to improve the search ability, convergence speed and execution efficiency of the PSO. The improved PSO algorithm can realize the accurate localization of beacon nodes by the following steps:

Step 1: Initialize the population. The number of particles is *Pnum*. Calculate the fitness of each particle in Equation (6). The individual extremum is denoted as *P_i_^t^*, and the global extremum is denoted as *P_g_^t^*.
  (6) $$f(t)=\frac{1}{H}\sum_{i=1}^{H}\left|\sqrt{{\left(x-x_{i}\right)}^{2}+{\left(y-y_{i}\right)}^{2}}-l_{i}\right|$$
  where, *H* is the number of effective buoys, (*x, y*) is the two-dimensional coordinate of beacon node, (*x_i_*, *y_i_*) is the coordinate of buoy, and *l_i_* is the distance from this beacon node to every buoy.
Step 2: Calculate the inertia weight with Equation (7), and update the position and velocity with Equations (8) and (9).
  (7) $$w(t)=(w_{\max}-w_{\min})\exp[-\frac{t^{2}}{{\left(k+t_{\max}\right)}^{2}}+w_{\min}]$$
  (8) $$V_{i}^{t+1}=V_{i}^{t}+c_{1}r_{1}(P_{i}^{t}-X_{i}^{t})+c_{2}r_{2}(P_{g}^{t}-X_{i}^{t})$$
  (9) $$X_{i}^{t+1}=X_{i}^{t}+V_{i}^{t+1}$$
  where, *t* is the iterations, *c_1_* and *c_2_* are the learning factors. *r_1_* and *r_2_* are the random numbers uniformly distributed in (0,1). $c_{1}r_{1}(P_{i}^{t}-X_{i}^{t})$ and $c_{2}r_{2}(P_{g}^{t}-X_{i}^{t})$ are the evolutions of a particle towards the local optimum and global optimum, respectively, and they can be regarded as the variation of velocity updated by theparticle in this iteration.
Step 3: Calculate the fitness of each particle and get the average fitness, then update the individual extremum $P_{i}^{t}$ and the global extremum $P_{g}^{t}$. Eliminate the particles whose fitness is larger than double average fitness.
Step 4: Judge whether it satisfies the condition: *P_g_^t^* < *ε* (*ε* is the threshold of position error) or *t = t_max_*. If one of the condition is satisfied, turn to Step 5, or *t* = *t* + 1, and turn to Step 2.
Step 5: Output the global optimal solution, and get the coordinates of the beacon nodes.

### 3.4. Localization of Unknown Nodes

Because the communication and computation ability of the underwater unknown nodes is limited, it is too difficult for them to perform the range-based localization algorithm. In this paper, by the proposed MP-PSO method, we use the temporal and spatial correlation of the underwater objects to realize the unknown nodes’ mobility prediction.

#### 3.4.1. The Calculation of Beacon Nodes’ Velocity

The range-based localization algorithm needs more energy and computation. In real practical applications, the number of beacon nodes in real networks is relatively less. These nodes have rather strong computational ability and more energy, so they can run the localization algorithm periodically to get their velocities of the previous time points.

**Figure 4:**
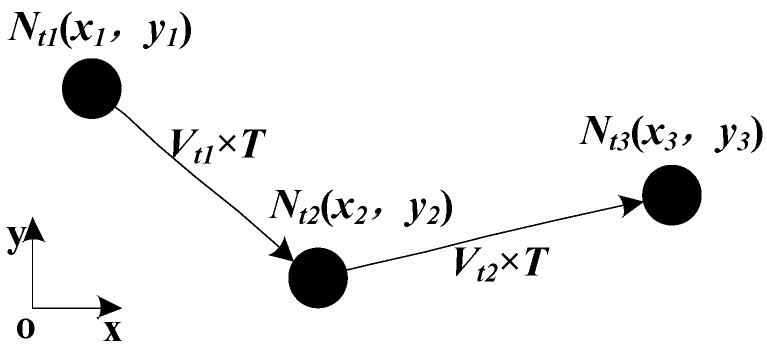
The calculation of beacon nodes’ velocity.

As shown in Figure 4, the beacon node N moves from *N_t1_* to *N_t2_* and then to *N_t3_*, and the position offsets of the two movements are *V_t__1_ × T* and *V_t__2_ × T* respectively. The coordinates of N at *t_1_* and *t_2_* can be obtained by the PSO algorithm, and then the velocity at *t_1_* for the beacon node can be calculated by Equation (10):

(10) $$v_{x}=\frac{x_{2}-x_{1}}{T};\;v_{y}=\frac{y_{2}-y_{1}}{T}$$

where *T* is the localization period, and the value of *T* may affect the localization result. Namely, if the value of *T* is too large, the estimation of the velocity of the beacon node may be imprecise. The instantaneity of velocity for the underwater object is strong, so if we use the velocity at a certain time to represent the velocity of a period in the past, it may cause large errors. On the other hand, if the *T* value is too small, the localization algorithm running frequency will be too high, which may cause more energy consumption and computational overhead.

#### 3.4.2. The Calculation of Unknown Nodes’ Velocity

**Figure 5:**
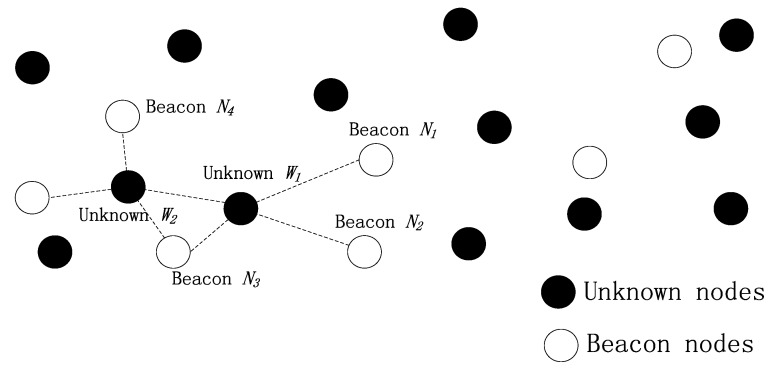
The calculation of unknown nodes’ velocity.

The beacon nodes broadcast a packet in the network after getting their velocities, and the packet contains the identity, velocity and time identification information. Time identification represents the moment of positioning for this node. Since all the nodes cannot complete their positioning at the same time, the referenced nodes selected should be in the same positioning round as the unknown nodes, that is to say they should have the same time identification. The unknown node *W* receives the packets from different beacon nodes, and it will sign the beacon node which has the same time identification as a referenced node. Finally the list of reference nodes will be set up, and it includes the identity, velocity, time identification information, and the received signal strength. The unknown node *W* can then calculate its mobile velocity with Equation (11):

(11) $$\{\begin{array}{c} v_{x}（w）=\sum_{i=1}^{M}\varsigma_{iw}v_{x}(i)\\ v_{y}（w）=\sum_{i=1}^{M}\varsigma_{iw}v_{y}(i) \end{array}$$

where, *M* is the number of referenced nodes, ς*_iw_* is the weight of referenced nodes, and it can be calculated by Equation (12):

(12) $$\varsigma_{iw}=\frac{r_{iw}}{\sum_{i=1}^{M}r_{iw}}$$

where, *r_iw_* is the signal strength received from the referenced node *i* by unknown node *W*. This method can decrease the errors of the referenced nodes which are far away from node *W*, and make the velocity of *W* more close to the real value. Considering the beacon nodes are relatively sparse in the underwater networks, we adopt a cooperative mechanism to calculate the velocity of the unknown nodes. The unknown nodes which already get their velocities can be taken as the new reference nodes. The principle of this method is shown in Figure 5.

As shown in Figure 5, the unknown node *W_1_* calculates its velocity using beacon nodes *N_1_*, *N_2_* and *N_3_*, and then *W_1_* broadcasts a packet containing the identity, velocity and time identification information, so the unknown node *W_2_* can calculate its velocity by using beacon nodes *N_3_*, *N_4_*, *N_5_* and the unknown node *W_1_* as the reference nodes.

The velocities of unknown nodes have relatively more errors, especially when the new referenced nodes are far away from the unknown node, it will cause larger error accumulation. In order to further decrease the error, the unknown node will distribute a confidence coefficient to each referenced node. We sort the referenced nodes according to their confidence coefficient from the biggest to the smallest, and select the first *M* nodes of among them to participate in the velocity calculation. According to Equations (11) and (12), we can know that the number of referenced nodes can affect the velocity calculation. If the value of *M* is too small, the velocity of the unknown nodes will be calculated by only a few nodes, which cannot take full use of the spatial correlation of underwater objects’ mobility. On the other hand, if the value of *M* is too large, the nodes which are far away from the unknown node will be involved in the velocity calculation, and it will cause large errors, so the value of *M* is determined by the number of all the nodes and the proportion of beacon nodes in the network. The confidence coefficient can be calculated by Equation (13):

(13) $$\eta_{iw}=\frac{r_{iw}}{r_{w\max}}-0.05k-0.1r$$

where, *r_iw_* is the signal strength received from reference node *i* by unknown node *W*, *r_wmax_* is the maximum of *r_iw_*, *k* is the number of unknown nodes which are involved in the calculation of the velocity for this reference node, and *r* is the times that the node is taken as the reference node. As an example in Figure 5, the values of *k* and *r* for beacon nodes are all 0. When we calculate the velocity of *W_2_*, the values of *k* and *r* of reference node *W_1_* are 0 and 1, respectively. When the node *W_2_* works as a reference node, the value of *k* is 1, and the value of *r* is 2.

#### 3.4.3. Updating of Unknown Nodes’ Location

After calculating the unknown nodes’ velocity, we can update their locations. In fact, we just need to store the velocity of each node and update the location when it is necessary. The location can be updated with Equation (14):

(14) $$\{\begin{array}{c} x\prime =x+\sum_{i=1}^{n}v_{xi}\cdot T\\ y\prime =y+\sum_{i=1}^{n}v_{yi}\cdot T \end{array}$$

where, *T* is the positioning period, and $\sum_{i=1}^{n}v_{i}\cdot t$ is the displacement between two positioning moments.

### 3.5. The Process of MP-PSO Method

As described above, the MP-PSO method is a kind of multi-steps localization algorithm, and the steps can be described as follows:

Step 1: Project the nodes into the plane which contains the surface buoys, and transform the three-dimensional localization problem into a two-dimensional localization.
Step 2: Locate the beacon nodes by using the range-based PSO algorithm.
Step 3: Calculate the velocity of beacon nodes according to the localization results at two instants.
Step 4: The unknown nodes sort their reference nodes in descending order according to the confidence value.
Step 5: If the number of reference nodes is more than *M*, then select the *M* reference nodes with larger confidence coefficients to calculate the velocity, if not, use all the reference nodes to calculate the velocity.
Step 6: Update the locations of the unknown nodes.

**Figure 6:**
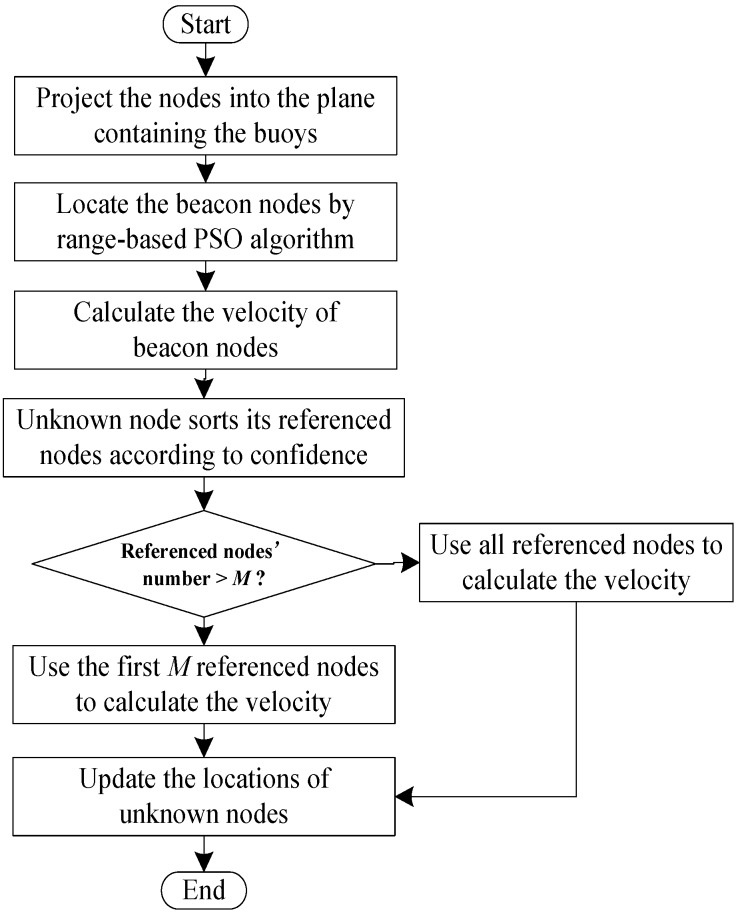
The process of MP-PSO algorithm.

The MP-PSO algorithm process is shown in Figure 6.

## 4. Simulations and Analysis

### 4.1. Parameter Setting 

We use MATLAB R2009a as the simulation platform to execute the algorithm in this paper. The settings of the simulation environment and related parameters are listed in Table 1.

**Table 1:** Simulation Parameters Setting.

Parameters	Values Setting
Deployment range	1000 *m* × 1000 *m*
Localization period	1 s
Communication radius of beacon nodes	200 m
Communication radius of unknown nodes	100 m
Transmission power	2 W(33 dBm)
Transmission frequency	50 kHz
Attenuation coefficient	2
Buoy number	20
Node number	200
*v*	N(1,(0.1)^2^)
*k_1_,k_2_*	N(π,(0.1π)^2^)
λ	N(3,(0.3)^2^)
*K_3_*	N(2π,(0.2π)^2^)
*K_4_,k_5_*	N(1,(0.1)^2^)

There are 20 surface buoys and 200 underwater nodes deployed in the monitoring area, the range of the area is 1000 m × 1000 m, and the depth of water is less than 100 m. We just investigate the moving state of the nodes driven by the water currents and waves in the seashore within 100 s after the initial deployment. In Table 1, the value of variables *v*, *k_1_–k_5_* and *λ* complies with the normal distribution, which can make the mobility of the nodes more close to the real environment.

**Figure 7:**
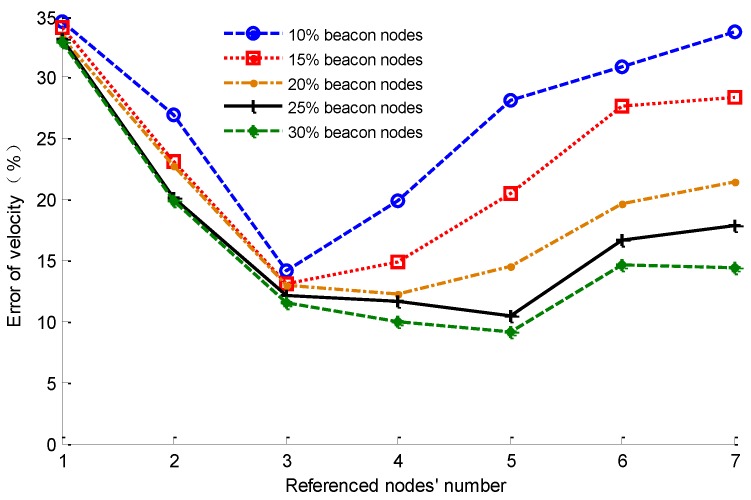
The relationship of velocity error and *M.*

According to the discussion in Section 3.4.2, the number of reference nodes can affect the localization result, so we need to select the best value of *M* by simulation testing. Keeping the parameter settings as in Table 1, by changing the proportion of beacon nodes to 10%, 15%, 20%, 25% and 30%, respectively, we can get the relationship of the average errors of the unknown nodes’ velocities with different values of *M* after 100 s. The result is shown in Figure 7.

From Figure 7, it can be seen that the velocity error first decreases, and then it begins to increase as the number of reference nodes increases. This is accord with the above analysis of the value of *M*. When the value of *M* is too large, the reference nodes whose confidences are smaller will participate in the velocity calculation, and may cause a large error accumulation. Figure 7 also shows that the optimal value of the number of reference nodes is related to the proportion of beacon nodes in the network. If the beacon node proportion is different, then the optimal value of *M* is also different. In the subsequent simulations, we select the value of *M* according to Figure 7.

### 4.2. Localization Results 

Keeping the settings of Table 1, we set the ratio of beacon nodes as 20%, and set the value of *M* as 4. We can simulate the proposed MP-PSO algorithm and analyze its positioning accuracy and positioning coverage rate. In this paper, we select the average error as an indicator to evaluate the performance of localization method [23], and it can be calculated as Equation (15):

(15) $$\bar{Error}=\sum_{i=1}^{K}\frac{\left|U_{i}\prime -U_{i}\right|}{K\times R_{a}}\times 100\%$$

where, *K* is the number of unknown nodes, *U_i_* is the real position of the unknown node, *U_i_'* is the located position of the unknown node, and *R_a_* is the communication radius of the unknown node. The error calculating as this manner denotes the proportion of the distance between the real position and the positioning position to the communication radius of the unknown node.

**Figure 8:**
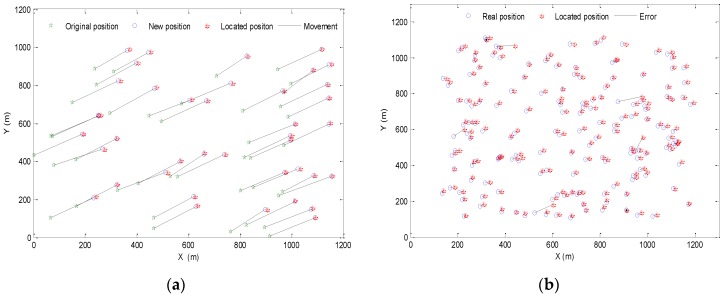
Localization results of (**a**) beacon nodes and (**b**) the unknown nodes.

The localization coverage rate, which denotes the proportion of the effectively located nodes to all the unknown nodes in the network is another important indicator to evaluate the localization algorithm. In this paper, we define the nodes whose error is less than 50% as the effectively located nodes. With the settings above, the localization results of the underwater nodes moving with the water current after 100 s are shown in Figure 8.

As shown in Figure 8a, the beacon nodes move with the water current for 100 s, we use the range-based PSO algorithm to locate them and the average error can be calculated, which is 3.26%, that is to say the absolute error is 6.52 m. This means the positioning accuracy of beacon nodes is not bad for this large-scale deployed underwater network. The higher positioning accuracy of the beacon nodes helps to make the error of prediction velocity of unknown nodes smaller, and it ensures the positioning accuracy of the whole algorithm to be relatively ideal. Figure 8b shows the comparison of the located and the real position after the unknown nodes move for 100 s, and it can be seen that some nodes’ error is too large, making them not effectively located nodes. The average error of the unknown nodes is 19.4% and the localization coverage rate is 97%.

**Figure 9:**
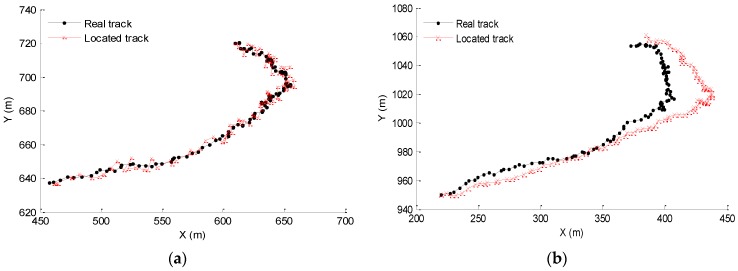
The comparison of (**a**) the beacon node’s track and (**b**) the unknown node’s track.

In the process of the nodes’ movement with the water currentw, we can record the position of a certain node at every moment within 100 s to plot its real movement track in 100 s, and we can also plot the located track according to the located position. Selecting the beacon node whose original coordinates are (452.9, 638.8) and the unknown node whose original coordinates are (219.7, 950.2) as the experimental subject, we can plot their real tracks and located tracks, which are shown in Figure 9.

Figure 9a shows the comparison between the real and located tracks for the beacon node within 100 s. It can be seen that the localization error almost does not change with the time. That is because the buoys’ coordinates are absolutely accurate, and there is no error accumulation in the range-based PSO algorithm. It also indicates that the PSO algorithm has better stability. Figure 9b shows that the error of unknown nodes increases gradually with time. This is because the localization of unknown nodes is a continuous prediction process, which may cause error accumulation and make the located tracks deviate from the real tracks as time goes on. While it is can be seen that the accuracy of the unknown nodes is relatively in the ideal range, the error is always limited within 50% in the process.

### 4.3. Impacts of Beacon Nodes’ Proportion on Localization Results

**Figure 10:**
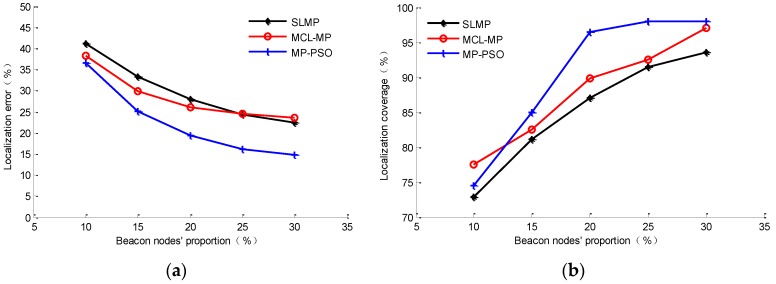
Impacts of the beacon node proportion on localization results: (**a**) on localization error; (**b**) on localization coverage.

Because the localization accuracy of beacon nodes is obviously higher than that of the unknown nodes, the proportion of beacon nodes can directly affect the localization results. Keeping the settings in Table 1 and changing the proportion of beacon nodes to 10%, 15%, 20%, 25% and 30%, respectively, the optimal value of the number of referenced nodes is selected according to Figure 7, and we can simulate and compare the localization results of the MP-PSO method, SLMP method and MCL-MP method after 100 s, respectively. The compared results are shown in Figure 10.

Figure 10a shows that MP-PSO method has an obvious localization accuracy advantage compared to the MCL-MP and SLMP methods. When the beacon node proportion is 20%, the average localization error of MP-PSO is decreased 8.7% compared to the SLMP method and 7.1% compared to the MCL-MP method. In addition, we can see that at the beginning of the increase of the proportion of beacon nodes, the localization accuracies of the three methods are all improved obviously. When the beacon node proportion is more than 20%, the decreasing trend of the localization error gradually becomes flat. Therefore, the effect of an excessive increase in the number of beacon nodes is insignificant, and it will only serve to increase the cost of the network, so it is necessary to keep a proper balance between the localization accuracy and the network cost in the deployment of a sensor network.

Figure 10b shows the state of localization coverage rate change with the proportion of beacon nodes for the three methods. As shown in the figure, when the proportion of beacon nodes is more than 20%, the localization coverage rate of the MP-PSO method can be higher than 95%, which is much better than the other two methods. That is because the MP-PSO method chooses the reference nodes with higher confidence coefficient to estimate the unknown nodes’ velocities, which can effectively decrease the localization error. In the MCL-MP method, the unknown nodes use the beacon nodes within their one-hop and two-hops range to predict their next position, so even if the proportion of beacon nodes increases more, inevitably some of the nodes cannot be located effectively due to the large localization error. In the SLMP method, the velocities of beacon nodes are estimated by the Durbin algorithm, and the error caused in this process is not related to the beacon node proportion, so its localization coverage rate does not increase as much as the MP-PSO method.

### 4.4. Impacts of Localization Period T on Localization Results

**Figure 11:**
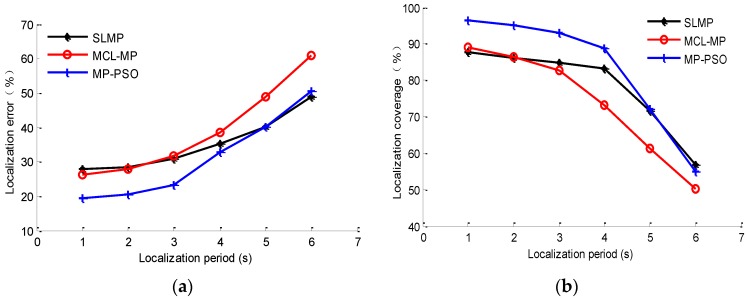
Impacts of localization period on localization results: (**a**) impact on localization error; (**b**) impact on localization coverage.

The localization period *T* is another factor which can affect the localization results. Keeping the settings in Table 1, choosing the proportion of beacon nodes as 20%, and changing the localization period from 1 s to 6 s, respectively, the localization results are as shown in Figure 11.

As shown in Figure 11a, as the localization period increases, the localization accuracy of the three methods decreases faster and faster. The accuracy of the MCL-MP method is affected by the localization period most. This is because this method realizes node localization by sampling prediction and filtering. The sampling range will increase as the localization period increases, which may cause larger localization errors. For the SLMP and MP-PSO methods, the bigger localization period will cause a larger displacement of the node between two positioning moments, and the node’s velocity changes all the time, so the error will increase when we use the velocity of one moment to represent the velocity of a period of time. The longer the time is, the larger the error will be. From Figure 11a, we can see that when the localization period is less than 4 s, the error of SLMP and MP-PSO methods will not increase much, but when it continues to increase more than 4 s, the accuracies decrease obviously, so the localization period of these two methods cannot be set too long. Considering the higher calculation efficiency of the MP-PSO method compared to the other methods, the small localization period is acceptable. In addition, Figure 11b shows that when the localization period is less than 4 s, the MP-PSO method has a far higher localization coverage rate than the other methods, and the localization coverage rate will not change much as the localization period changes, which proves that the proposed method has a better stability in this stage.

### 4.5. Impacts of Nodes’ Velocity v on Localization Results

**Figure 12:**
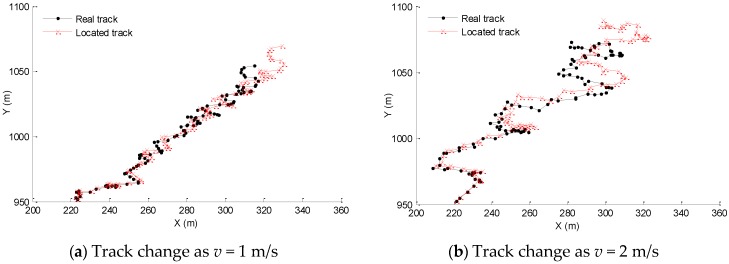

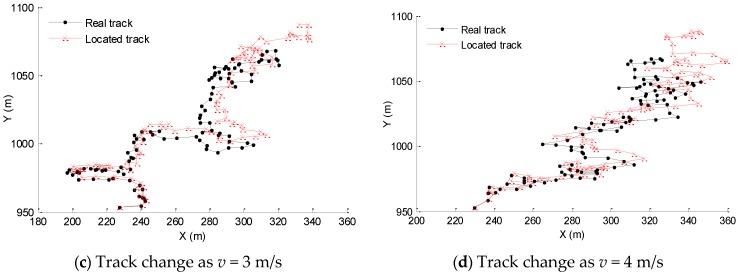
Impacts of nodes’ velocity on localization results.

Keeping the parameter settings in Table 1, setting the proportion of beacon nodes as 20%, and changing the nodes’ velocity, randomly taking the unknown node whose original coordinate is (229.78, 953.04) as example, the comparisons of tracks under different velocities are shown in Figure 12.

As shown in Figure 12, with different velocities, the deviation of the located and real position of the unknown node becomes large as the time goes on, and this is accord with the analysis of the localization error of unknown nodes above. At the same time, as the velocity increases, we can see that the localization error increases gradually. From Figure 12a to Figure 12d, with the velocity increasing from 1 m/s to 4 m/s, the range of the nodes movement increases as well, and the deviation of the located trajectory from the actual trajectory also increases gradually. That is because when the velocity increases, the distance of nodes’ movement between two positioning moments will increase with the same localization period, so the relevant localization error will increase as well. Therefore, this method is suitable for the underwater environment in which the water flow is steady and the mobility of nodes is not very fast. Considering that underwater WSNs are mainly used in the seashore environment currently, where the water is shallow and the nodes’ velocity is relatively slow, this method has better applicability in underwater WSNs with mobile nodes.

### 4.6. Analysis of the Energy Consumption

**Figure 13:**
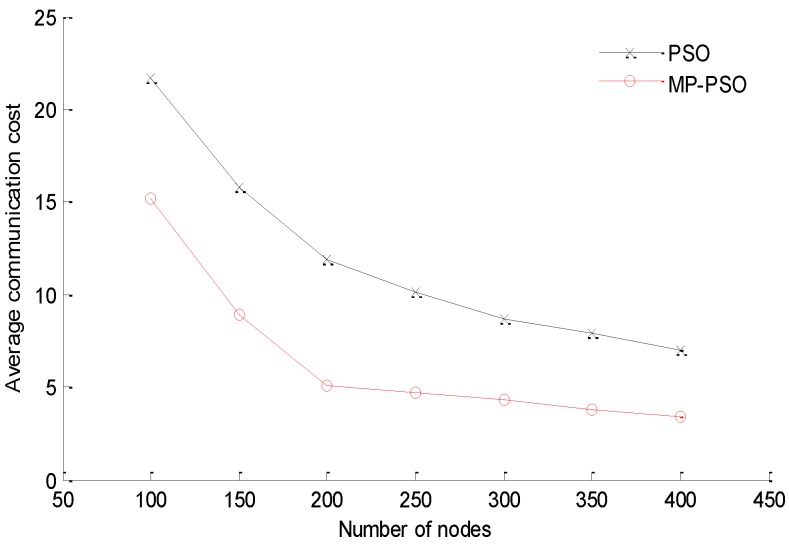
The comparison of energy consumption.

Energy consumption is another important indicator to evaluate the localization methods, and it is mainly the result of the communication activities among the nodes. The communication consumption can be described by the average communication cost, which is defined as the ratio of the overall messages exchanged in the network to the number of effectively localized sensor nodes. Keeping the parameter settings in Table 1, setting the beacon nodes’ proportion as 20% and the value of *M* as 4, we can simulate and analyze the average communication cost of the range-based PSO and MP-PSO methods when the number of the whole network nodes changes to 100, 150, 200, 250, 300, 350 and 400, respectively. The result is shown in Figure 13.

Figure 13 clearly shows that with an increasing number of nodes in the network, the average communication cost of PSO and MP-PSO all decrease at a ratio of about 10% before the number of nodes reaches 200. This is because as the number of nodes increases, the number of effectively located nodes increases drastically. Further, when the number of nodes increases to more than 200, the rates of change of the average communication cost for the two methods all become slow, and ultimately stabilize at some small value. At this stage, the droop rates of PSO and MP-PSO are about 2.25% and 0.6% respectively. Meanwhile, Figure 13 also shows the mobility prediction scheme can greatly reduce the average communication cost of the network. When the number of nodes is less than 250, the average communication cost of MP-PSO is approximate 6 lower than PSO, and when the number of nodes is more than 250, the average communication cost of MP-PSO is approximately 4 lower than PSO. The decrease of average communication cost of the MP-PSO method is about 50% compared to the PSO method. This is because in the mobility prediction procedure, the unknown nodes need not establish communication links with every beacon node and the buoys. In fact, the unknown nodes only need to communicate with several reference nodes to predict their next location. The proposed scheme can effectively reduce the overall data package exchange in the network. Accordingly, the average communication cost of the network will be reduced. This is quite significant for UWSNs with limited bandwidth and energies.

## 5. Conclusions

Focusing on the problems of underwater WSNs, like sparse deployment and mobility of the nodes driven by water currents, a multi-step localization method based on mobility prediction and PSO algorithm is proposed. The beacon nodes are located by the range-based PSO algorithm and the velocities can be calculated by using the localization results, and then the unknown nodes are located by the mobility prediction based on the estimation of the spatial correlation of underwater objects’ mobility. The simulation results show that this method can provide highly accurate localization of beacon nodes, and the positioning accuracy and positioning coverage rate can be kept at a better level. Compared to the SLMP method which uses the Durbin algorithm to estimate the beacon nodes’ velocity and the MCL-MP method which is based on sampling prediction and filtering, with the same localization period, the MP-PSO method obviously has higher localization accuracy and coverage rate. With the same proportion of beacon nodes, the localization result of MP-PSO also has obvious advantages with a shorter localization period as well. In addition, the energy consumption can be reduced by the proposed mobility prediction scheme, so the proposed localization method of mobile nodes in UWSNs can be applicable in the fields of military defense, environmental monitoring and animal tracking, *etc.*
